# Decoupling metasurface parameters for independent Stokes polarization control via generalized lattice

**DOI:** 10.1038/s41377-025-02084-6

**Published:** 2026-01-04

**Authors:** Zhi Cheng, Zhou Zhou, Zhuo Wang, Yue Wang, Changyuan Yu

**Affiliations:** 1 Department of Electrical and Electronic Engineering, The Hong Kong Polytechnic University, Hong Kong SAR, China; 2 Department of Electrical and Computer Engineering, and NUS Graduate School, National University of Singapore, Singapore, 117583 Singapore; 3 Guangdong Provincial Key Laboratory of Nanophotonic Functional Materials and Devices, School of Optoelectronic Science and Engineering, South China Normal University, Guangzhou, 510006 China

**Keywords:** Metamaterials, Micro-optics

## Abstract

The ability to achieve comprehensive control over all Stokes parameters, including both the state of polarization (SoP) and the degree of polarization (DoP), is fundamental to advancements in quantum optics, imaging, and optical communications. While metasurfaces have demonstrated remarkable capabilities in polarization manipulation, existing designs typically rely on locally periodic unit cells and deterministic phase profiles, limiting their flexibility in controlling both SoP and DoP simultaneously. Here, we introduce the generalized lattice approach for metasurface design, which enables the decoupling of structural parameters from the full-Stokes polarization response. Our approach introduces a spatially global but structurally disordered arrangement, constructed on a generalized lattice framework. This framework enables the flexible placement of an arbitrary number and type of meta-atoms within a generalized lattice, where the relative quantity ratios among different meta-atoms serve as a new design degree of freedom. This decoupling enables the azimuthal and elevation angles of the SoP on the Poincaré sphere to be governed by the in-plane rotation and size of individual meta-atoms, while the DoP is controlled independently via the quantity ratio. This establishes a direct and analytically tractable mapping between metasurface geometry and polarization space, offering new physical insights into metasurface-based polarization control. A computationally efficient algorithm optimizes the metasurface arrangement, achieving a polarization similarity (evaluated by Stokes Euclidean Distance) of 0.93 in theory and 0.90 in experiment. Our findings demonstrate that the generalized lattice approach provides an effective and versatile route to full-Stokes polarization control with greater flexibility than conventional metasurface designs.

## Abstract (web summary)

A disordered metasurface based on a generalized-lattice strategy enables uniform arrangement of meta-atoms with arbitrary types and quantity ratios, decoupling structural design from all Stokes parameters.

## Graphical abstract

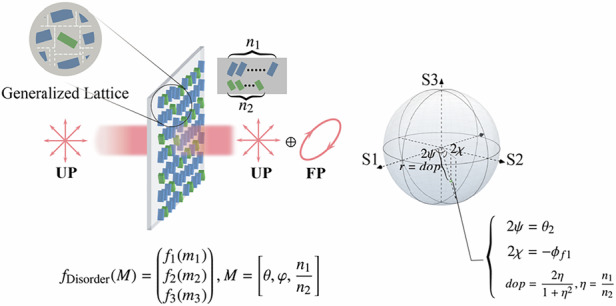

## Introduction

The capability to precisely manipulate the full-Stokes parameters of light, encompassing both the state of polarization (SoP) and degree of polarization (DoP), is a cornerstone of photonics research and development. In deterministic systems, polarization refers to the orientation of the electric field vector, which defines the SoP. In stochastic systems, polarization evolves over time, giving rise to light that may be unpolarized or partially polarized. This variability is quantitatively described by the DoP, which measures the fraction of light that remains polarized. Partially polarized light is thus a combination of fully polarized (dominant polarization) and unpolarized components, with the DoP indicating the relative contribution of the polarized component.

Controlling SoP and DoP is vital for applications such as optical communication^1–3^, quantum optics^4–7^, imaging^8,9^, remote sensing^10^, and spectroscopy^11–13^. SoP manipulation is commonly achieved through wave plates, which introduce controlled phase shifts between orthogonal polarization components. On the other hand, DoP modulation typically involves converting fully polarized or unpolarized light into a desired partially polarized state. Achieving this requires differential transmission between orthogonal polarization states. This is often implemented experimentally using time-varying optical retarders or wavelength-dependent wave plates. Simultaneous control over both SoP and DoP, however, presents significant engineering challenges. Traditional approaches for such control often rely on complex and bulky optical setups, which can impede the integration and miniaturization of photonic devices. These limitations highlight the need for more compact and efficient solutions capable of fully manipulating the polarization properties of light.

**Fig. 1:**
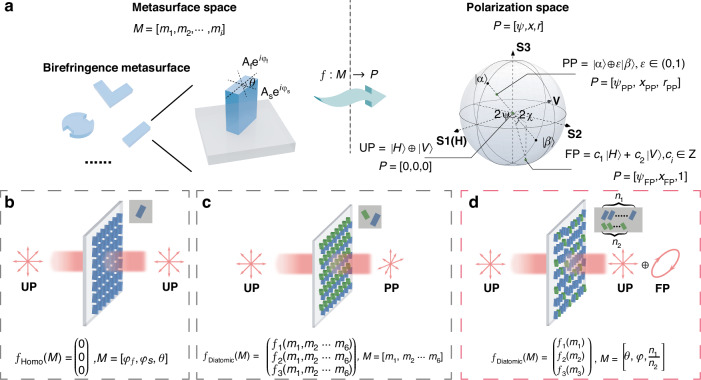
**Conceptual diagram of this work.** **a** The mapping between metasurface parameter space and polarization control space. The metasurface parameters include the complex transmission coefficients of the fast and slow axes, as well as the rotation angle. A point on or inside the Poincaré sphere is defined by the azimuth *ψ*, elevation angles *χ*, and radius *r*. The symbol ⊕ represents the incoherent superposition of light. PP partially polarized, UP unpolarized, FP fully polarized. **b** The homogeneous metasurface with one meta-atom in a unit cell cannot convert unpolarized light into partially polarized light. **c** The diatomic metasurface with two meta-atoms in a supercell converts unpolarized light into partially polarized light. **d** The proposed disordered metasurface could partially polarize natural light with the independent control of the dominant full-polarized light and DoP

Recently, metasurfaces—comprising arrays of nanoscale structures—have garnered significant attention as flat optical devices capable of manipulating light’s amplitude, phase, and polarization at subwavelength scales. These capabilities have catalyzed advancements in fields such as imaging^14–19^, polarization control^20–36^, quantum optics^37–41^, and communications^42–45^. Importantly, metasurfaces facilitate the miniaturization and integration of photonic devices, offering enhanced functionality within compact platforms. As shown in Fig. 1a, in the context of polarization control, a mapping relationship between the metasurface design parameter space and the polarization control parameter space can be established through careful design. The homogeneous metasurface composed of birefringent meta-atoms, shown in Fig. 1b, functions as a nano-waveplate widely used in polarization modulation^46–48^. When the transmittance is set close to unity, the system becomes effectively lossless, yielding a unitary Jones matrix. This allows the metasurface to convert one SoP into another by redistributing phase delay between orthogonal polarization components. However, in dynamic systems with incoherent light, time-averaging equalizes intensity differences between these components, rendering this approach unsuitable for controlling the DoP. Based on the concept of a weak coupling region^25,26^, we adjust the gap distance between the two types of meta-atoms. This adjustment induces far-field radiation interference in the diatomic structure shown in Fig. 1c. This design could provide at least six degrees of freedom, enabling the realization of any arbitrary Jones matrix for polarization conversion^20–25^. The flexibility of this method allows for the creation of non-unitary Jones matrices, which generate intensity differences between orthogonal states in a time-varying system. However, this results in an intrinsic coupling between the metasurface design parameters, specifically the dominant SoP and the DoP within the polarization space, as shown in Fig. 1c. This relationship can be further understood using the phasor diagram presented in Fig. S1. Wang et al. recently tackled this challenge by introducing an inverse-designed meta-atom, optimized through topological methods, capable of converting unpolarized light into partially polarized light^32^. However, the complex geometries of these optimized structures require high-precision fabrication, which presents significant challenges for practical implementation.

In this study, we propose a novel generalized lattice approach to design metasurfaces that enable independent control over the SoP and DoP. This proposed metasurface, illustrated in Fig. 1d, is composed of two types of birefringent meta-atoms uniformly distributed across its surface. By leveraging far-field interference effects, we introduce polarization-dependent loss to precisely modulate the DoP. The DoP is controlled by the differential conversion of orthogonal polarization components, determined by the ratio of different meta-atoms. SoP control is achieved by adjusting the azimuth and elevation angles on the Poincaré sphere through the rotation and phase difference of individual meta-atoms. To achieve this, we developed a placement algorithm based on greedy search and 2D bin-packing to design a non-periodic metasurface with disordered features under the generalized lattice framework. The disordered structure effectively mitigates higher-order diffraction introduced by the non-periodic configuration. This framework allows arbitrary types of meta-atoms to be distributed with adjustable spatial ratios, enabling systematic access to all Stokes parameters. Through a combined theoretical and experimental approach, we demonstrate independent control over all three degrees of freedom of polarization states on the Poincaré sphere—latitude, longitude, and radial position—via precise manipulation of meta-atom size, orientation, and quantity ratio within the metasurface architecture. It is also shown that the design possesses a degree of tolerance, allowing different meta-atom combinations to function effectively within the same framework. Furthermore, we demonstrate the flexibility of this design approach in enabling arbitrary polarization state conversion applications.

## Results

**Fig. 2:**
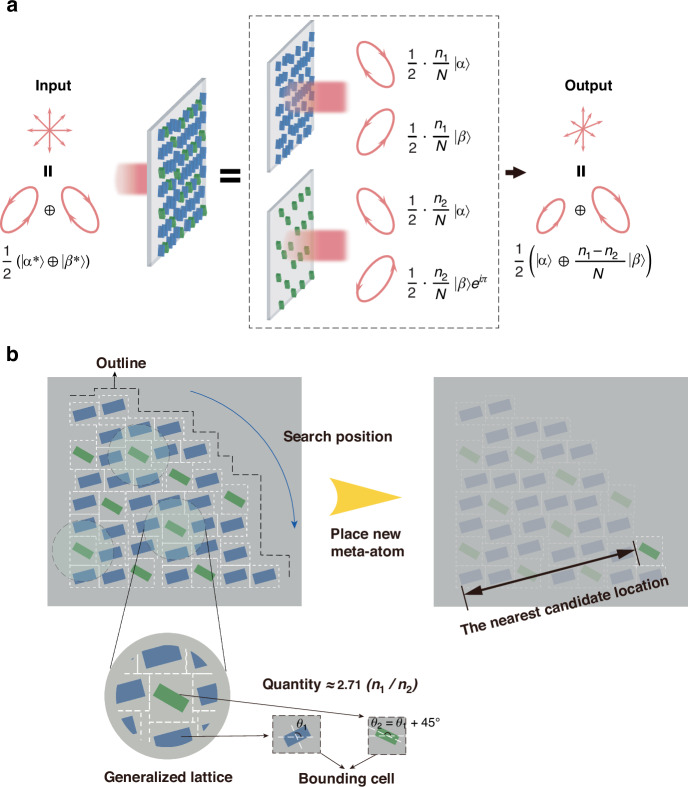
**Principle of Stokes parameter decoupling and the metasurface arrangement process.** **a** The physical mechanism of partially polarizing natural light with the independent control over the SoP and DoP using metasurfaces. **b** The process of placing meta-atoms using the proposed 2D bin-packing strategy, illustrated with a case where two meta-atoms are placed with a quantity ratio of 2.71. The white dashed boundaries represent the bounding cell used to define the weak coupling region

The physical mechanisms that simultaneously and independently control DoP and SoP using metasurfaces are illustrated in Fig. 2a. Unpolarized light is often mathematically represented as a superposition of two orthogonal polarization states, simplifying the description by ignoring the random phase relationships inherent in unpolarized light. This representation allows the analysis to focus on the polarization state transformation while abstracting away the complexity of random phase variations. Each metasurface can convert ($|{\alpha }^{* }\rangle$, $|{\beta }^{* }\rangle$) into ($|\alpha \rangle$, $|\beta \rangle$), with distinct phase shifts for the respective states. This results in constructive interference for the $|\alpha \rangle$ state and destructive interference for the $|\beta \rangle$ state. When the two metasurfaces differ in their proportional contribution, the degrees of constructive interference for $|\alpha \rangle$ and destructive interference for $|\beta \rangle$ are no longer balanced, leading to an unequal ratio of $|\alpha \rangle$ and $|\beta \rangle$ in the final incoherent light output. In essence, differential conversion between $|\alpha \rangle$ and $|\beta \rangle$ is achieved, resulting in partially polarizing the unpolarized light. In what follows, the derivation of the necessary Jones matrices, the mechanism for controlling DoP, and the design methodology for the metasurfaces are discussed.

We begin with the birefringent meta-atoms, which are commonly used for polarization control. The behavior of these meta-atoms is described by the following Jones matrix:

1 $${J}_{{atom}}=R(\theta )\left(\begin{array}{cc}{A}_{f}{e}^{i{\varphi }_{f}} & 0\\ 0 & {A}_{s}{e}^{i{\varphi }_{s}}\end{array}\right)R(-\theta )$$

This Jones matrix accounts for the phase retardation along the fast and slow optical axes, denoted by ${\varphi }_{f}$ and ${\varphi }_{s}$, respectively, with *A*_*f*_ and *A*_*s*_ representing the transmittances along these axes. The rotation matrix *R(θ)*, defined $$R(\theta )=\left[\begin{array}{cc}\cos (\theta ) & -\sin (\theta )\\ \sin (\theta ) & \cos (\theta )\end{array}\right]$$, describes the orientation of the birefringent axes with respect to the incident polarization. With transmittances close to unity, the system becomes effectively lossless, yielding a unitary Jones matrix. To introduce differential transmission between orthogonal states, a non-unitary Jones matrix is required, which can be constructed as a sum of unitary matrices.

On the other hand, the Jones vectors of an arbitrary orthogonal polarization pair can be expressed in terms of azimuth angle *ψ* and elevation angle *χ* on the Poincaré sphere as:

2 $$|\alpha \rangle =R(\psi -{45}^{\circ })\left(\begin{array}{l}{e}^{-i\chi }\\ {e}^{i\chi }\end{array}\right),|\beta {\rm{\rangle }}=R(\psi -{45}^{\circ })\left(\begin{array}{l}{e}^{-i\chi }\\ -{e}^{i\chi }\end{array}\right)$$

Here, $|\alpha \rangle$ is located at coordinates (2*ψ*, 2*χ*) on the Poincaré sphere, while $|\beta \rangle$ is positioned at (2*ψ* − 180°, −2*χ*), symmetrically opposite about the origin. It is important to note that, in the polarization states ($|\alpha \rangle$, $|\beta \rangle$), the *x*- and *y*-components are complex conjugates, except for the negative sign in $|\beta \rangle$. This indicates that by applying a phase delay to the *x*- and *y*-components, as described by the Jones matrix in the form of Eq. (1), it is possible to convert between the polarization states *(*$\left|\alpha \right\rangle$, $\left|\beta \right\rangle$*)* and their conjugate states ($|{\alpha }^{* }\rangle$, $|{\beta }^{* }\rangle$) (Supplementary Information Note II). Therefore, the Jones matrix for converting the orthogonal conjugate states can be expressed in a form similar to Eq. (1):

3 $${J}_{1}=R(\psi -{45}^{\circ })\left(\begin{array}{cc}{e}^{-2i\chi } & 0\\ 0 & {e}^{2i\chi }\end{array}\right)R({45}^{\circ }-\psi )$$

This Jones matrix converts the conjugate orthogonal states ($|{\alpha }^{* }\rangle$, $|{\beta }^{* }\rangle$) into ($|\alpha \rangle$, $|\beta \rangle$) while introducing the same global phase. This means that there is no relative phase shift between the two states. Another Jones matrix can be constructed to achieve the conversion between these two pairs of conjugate orthogonal states with a relative phase difference of π (see Supplementary Information Note II). The Jones matrix for this transformation can be expressed as:

4 $${J}_{2}=R(\psi )\left(\begin{array}{cc}1 & 0\\ 0 & -1\end{array}\right)R(-\psi )=R(\psi -45)\left(\begin{array}{cc}0 & 1\\ 1 & 0\end{array}\right)R(45-\psi )$$

This matrix flips the *x*- and *y*-components of the Jones vector in Eq. (2). The negative sign in the *y*-component of the $|\beta \rangle$ state introduces a global phase of π during the conjugate state conversion process. By combining the Jones matrices from Eqs. (3) and (4) with different coefficients, differential transmission between orthogonal polarization states can be achieved, as shown in Fig. 2a. The resulting composite Jones matrix can be expressed as follows:

5 $$J={J}_{1}+{J}_{2}=\frac{{n}_{1}}{N}R\left(\psi -{45}^{\circ }\right)\left(\begin{array}{cc}{e}^{-2i\chi } & 0\\ 0 & {e}^{2i\chi }\end{array}\right)R\left({45}^{\circ }-\psi \right)+\frac{{n}_{2}}{N}R\left(\psi \right)\left(\begin{array}{cc}1 & 0\\ 0 & -1\end{array}\right)R\left(-\psi \right)$$

To ensure energy conservation, the sum of the coefficients must be equal to unity. Therefore, the coefficients are written in the form of *n*_*i*_*/N*, where *i* = 1, 2, and *N* = *n*_*1*_ + *n*_*2*_. The state $|{\alpha }^{* }\rangle$ is fully converted into $|\alpha \rangle$ with an amplitude of 1/2. However, the conversion between $|{\beta }^{* }\rangle$ and $|\beta \rangle$ undergoes destructive interference due to the π-phase delay, resulting in an amplitude of (*n*_1_ − *n*_2_)/2*N*. Consequently, the transmission matrix for converting ($|{\alpha }^{* }\rangle$, $|{\beta }^{* }\rangle$) into ($|\alpha \rangle$, $|\beta \rangle$) is given by:

6 $${J}_{t}=\left(\begin{array}{cc}{t}_{{\alpha }^{* }\alpha } & {t}_{{\beta }^{* }\alpha }\\ {t}_{{\alpha }^{* }\beta } & {t}_{{\beta }^{* }\beta }\end{array}\right)=\left(\begin{array}{cc}1 & 0\\ 0 & \frac{{n}_{1}-{n}_{2}}{N}\end{array}\right)$$

To determine the DoP in the time domain, it is necessary to apply a time-averaged operation to account for the random phase in unpolarized light. The intensity of the outgoing light can then be expressed as:

7 $$\begin{array}{l}\langle {I}_{{out}}\rangle =(1-{t}_{{\beta }^{* }\beta }^{2})\langle \alpha \rangle +{t}_{{\beta }^{* }\beta }^{2}(\langle \alpha \rangle +\langle \beta \rangle )\\ =(1-{t}_{{\beta }^{* }\beta }^{2}){FP}+2{t}_{{\beta }^{* }\beta }^{2}{UP}\end{array}$$

where the brackets <> denote to time average. According to the definition of DoP, let *η* = *n*_*1*_*/n*_*2*_, it is calculated as:

8 $$p=\frac{1-{t}_{{\beta }^{* }\beta }^{2}}{1+{t}_{{\beta }^{* }\beta }^{2}}=\frac{2\eta }{1+{\eta }^{2}}$$

As a result of the preceding derivation, a one-to-one correspondence between the structural parameters of the metasurface and the coordinates on the Poincaré sphere has been established. A UP light can be partially polarized by the proposed metasurface, as analyzed using a Mueller matrix provided in Supplementary Information Note III. The polarization orientation, represented by ${S}_{1}$, ${S}_{2}$, and ${S}_{3}$, can be mapped to the azimuth and elevation angles on the Poincaré sphere, which is governed by the rotation angle and phase delay of the meta-atoms, as described by Eq. (5). Meanwhile, the DoP, corresponding to the radius in the Poincaré sphere, is controlled by the ratio of the two types of meta-atoms (*n*_*1*_/*n*_*2*_) within the disordered metasurface. This decoupled control allows for independent tuning of the polarization state’s orientation and its purity, enabling full manipulation of all relevant Stokes parameters.

To achieve the Jones matrix in Eq. (5), we propose a disordered metasurface composed of various types of meta-atoms. These adjacent meta-atoms are arranged with appropriate gap distances in the weak coupling regime. In this case, the Jones matrix is the summation of the matrices of each meta-atom, represented in the following form:

9 $${J}_{{DM}}=\mathop{\sum }\limits_{i=1}^{M}\frac{{n}_{i}}{N}{J}_{{atom}}^{i}$$

where *n*_*i*_ (*i* = 1, 2…*M*) represents the number of each meta-atom, and *N* denotes the total number of atoms. When *M* = 2 and *n*_*1*_ = *n*_*2*_, the metasurface degenerates into the simpler diatomic design shown in Fig. 1c, where a periodic unit cell within wavelength scale is constructed across the metasurface. However, as detailed in Supplementary Information Note I, this configuration makes it infeasible to decouple the design parameters needed for independent control of the SoP and DoP using a Jones matrix in the form of Eq. (5). This leads to scenarios where *n₁* ≠ *n₂* or *M* > 3, requiring more than 2-meta-atoms to be placed in a supercell. Restricting the lattice size to a subwavelength scale inherently limits the number of meta-atoms that can be placed within the lattice, as weak coupling and local effects must be maintained. Literature indicates that this number is typically constrained to 2–4 meta-atoms, which proves insufficient for achieving arbitrary parameter decoupling. On the other hand, incorporating a larger number of meta-atoms within a single lattice inevitably increases the lattice size beyond the wavelength, leading to strong higher-order diffraction modes. As detailed in Supplementary Information Note IV, such periodic designs result in significant energy loss and unintended polarization conversion due to higher-order diffraction and phase gradients.

To achieve the desired arrangement of meta-atoms, we developed a placement strategy based on a two-dimensional bin-packing problem. It ensures that the metasurface satisfies two key conditions: (1) each type of meta-atom is uniformly distributed across the entire surface, and (2) the overall quantity ratio of the two types of meta-atoms ($n_{1}/n_{2}$) matches the target value. Together, these conditions guarantee that within any localized region, defined by a generalized lattice, the quantity ratio of meta-atoms approximates the target value, ensuring both local and global uniformity.

Figure 2b illustrates the process of placing a single meta-atom using the proposed strategy. The detailed flowchart and step-by-step description of this are provided in the Supplementary Information Note V. Each meta-atom is enclosed within a bounding cell, a rectangular region that defines its effective working area. The bounding cell size is determined by the weak coupling region, which our empirical calculations show typically spans about 1/10 of the operating wavelength. A bounding cell that is too small leads to strong near-field interactions, while an excessively large cell increases the distance between meta-atoms, introducing noticeable phase differences in the far field. By maintaining sufficient spacing between bounding cells, the algorithm avoids strong coupling while preserving far-field coherence.

To evaluate the uniformity of meta-atom placement, a generalized lattice is defined as a circular region approximately one wavelength in diameter. This lattice represents the effective evaluation area where the local quantity ratio of type I and type II meta-atoms is assessed against the target value ($n_{1}/n_{2}$). The uniform distribution of meta-atoms across the metasurface is achieved by ensuring that the quantity ratio within each generalized lattice matches the target value, enabling far-field coherence and consistent performance. In the Fig. 2b, positions are searched along the outline, and a meta-atom with its bounding cell is placed at the closest point. This placement ensures that the local quantity ratio within the generalized lattice progressively approaches the target value, maintaining uniformity across the metasurface.

**Fig. 3:**
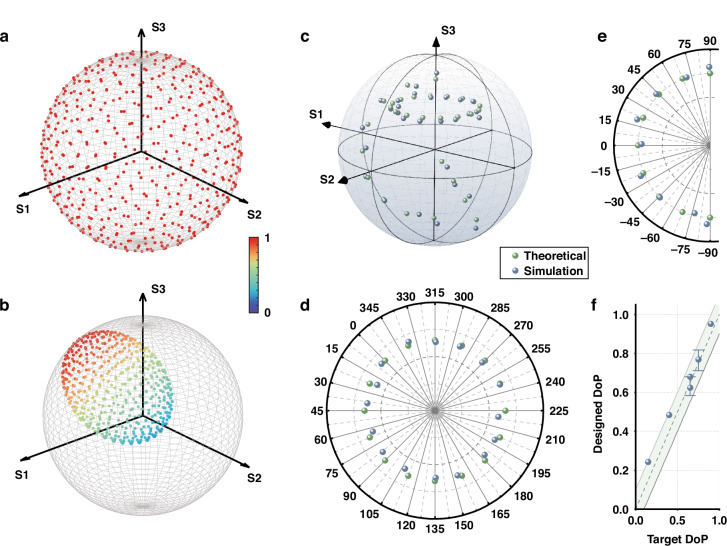
**Demonstration of independent control of the azimuth and elevation angles of the dominant polarization state and the DoP.** **a** 500 uniformly sampled points on the Poincaré sphere, simulating natural light with arbitrary polarization states. **b** 500 points distributed inside and on the surface of the Poincaré sphere, representing partially polarized light after metasurface conversion. Color indicates light intensity. Theoretical parameters in this conversion are 2*ψ* = 300°, 2*χ* = 45°, and DoP = 0.65, respectively. **c** Evolution of the polarization on the Poincaré sphere along the latitude at 2*χ* = 45° with DoP = 0.65, longitude at 2*ψ* = 45° with DoP = 0.75, and radius at 2*χ* = −60°, 2*ψ* = 225°, respectively. The green dots represent the target polarization, while the blue dots represent the simulation results. **d** The top view of (**c**) illustrates the polarization evolution along the latitude. **e** The side view of (**c**) illustrates the polarization evolution along the longitude. **f** The DoP values of all calculation points. The scatter points are along the radius, and the other two error bars are along the longitude and latitude, respectively. The green shaded area represents the range of DoP error ±0.1

We validate the proposed concept by demonstrating full control of the SoP and DoP across the entire Poincaré sphere through simulations conducted using the Finite-Difference Time-Domain (FDTD) method, as shown in Fig. 3. For fully polarized light, the polarization state is represented by points on the surface of the Poincaré sphere, while partially polarized light is depicted by points within the sphere. Complete polarization control requires the manipulation of three parameters on the Poincaré sphere: azimuthal angle, elevation angle, and radius, which correspond to the Stokes parameters ${S}_{1},{S}_{2},{S}_{3}$, and the DoP. By independently varying these parameters, we demonstrate precise control over the latitude, longitude, and radius of the Poincaré sphere, allowing for arbitrary manipulation of partially polarized light.

In the simulation, 500 points are uniformly sampled across the surface of the Poincaré sphere as shown in Fig. 3a, with near-equal Euclidean distances between adjacent points, to represent the input of unpolarized light. These discrete points also reflect the randomness and statistical nature of unpolarized light, representing the distribution of polarization states over time. This approach effectively maps the polarization states that occur over a period of time onto the Poincaré sphere, providing a comprehensive representation of the characteristics of unpolarized light. Similarly, for partially polarized light, this method is also applied, as shown in Fig. 3b. The random phase component inherent in unpolarized light is neglected, as it does not influence the results in time-averaged incoherent superposition calculations. The polarization states corresponding to the sampled points are converted by the designed metasurface, resulting in a non-uniform distribution of Stokes vectors across both the surface and interior of the Poincaré sphere, as shown in Fig. 3b. This unique distribution on the Poincaré sphere serves as a distinctive fingerprint of partially polarized light, encapsulating its statistical nature and providing profound insights into its transformation through the metasurface. The incoherent sum of these transformed polarization states yields the DoP and the dominant SoP.

Figure 3c–f demonstrates the evolution of polarization states on the Poincaré sphere, showcasing how the azimuth, elevation angles, and DoP can be independently controlled. The latitude and longitude lines on the Poincaré sphere pass through the polarization state at 2*χ* = 45° and 2*ψ* = 45°, respectively. These polarization states are achieved by varying the size or orientation of two types of meta-atoms. Only half of the longitude is depicted, as the other half can be obtained by rotation of the meta-atoms. To enhance visualization clarity, the dominant SoP is chosen as (2*χ* = −60°, 2*ψ* = 225°), located in the southern hemisphere, when demonstrating DoP control through adjustments to the meta-atom quantity ratio. For the latitude data set, the DoP is set to 0.65, while for the longitude data set, it is set to 0.75, corresponding to quantity ratios of 2.714 and 2.214, respectively. In the metasurface design, the target DoP values are approximated by quantity ratios of 19:7 and 31:14 for the two types of meta-atoms. The arrangement coordinates and structural parameters of the employed meta-atoms, along with the database of their propagation phase modulation and size parameters, are detailed in Supplementary Information Note VI. In Fig. 3, the green points represent the target polarization states on the Poincaré sphere, while the blue points represent the simulation results. The latitude and longitude evolution data sets are also shown in Fig. 3d and e, with top-view and side-view perspectives, respectively. The average error in the elevation angle is 2.65°, while the average error in the azimuth angle is 2.98°, demonstrating the accuracy of this method in controlling the polarization state. In Fig. 3f, error bars indicate the range of simulated DoP values for the latitude and longitude data sets, with the central sphere symbol representing the average DoP. The green shaded area corresponds to a DoP error range of ±0.1, while nearly all data points fall within the tighter range of ±0.05. As the elevation angle increases, we observe a rise in error. This is attributed to the limited size of the meta-atom database (see Supplementary Information Note VI) and the relatively large size difference between the two types of meta-atoms in the high-latitude regions. This discrepancy complicates the task of finding an appropriate spacing to satisfy both weak coupling and coherence conditions. The mismatch between the propagation phase of the selected meta-atoms and the target phase could also contribute to the observed errors. Expanding the meta-atom database could help mitigate both issues and reduce the error.

**Fig. 4:**
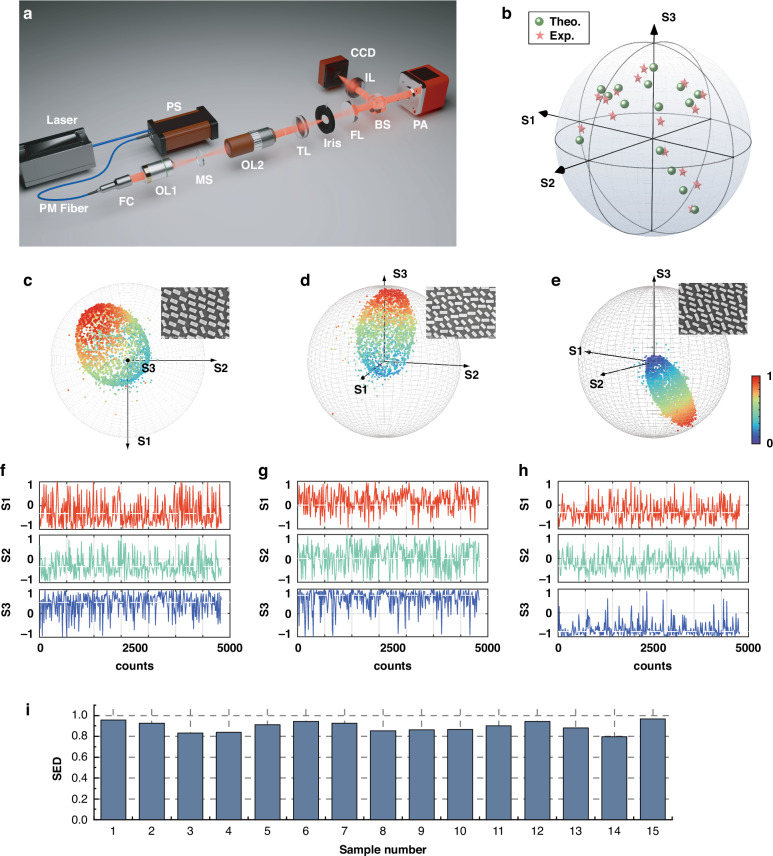
**Experimental validation of the independent control of all Stokes parameters.** **a** Experimental setup employed for measuring partially polarized light. PS polarization scrambler, PM fiber polarization-maintaining fiber, FC fiber collimator, OL objective lens, MS metasurface, TL tube lens, FL Fourier lens, IL imaging lens, BS beam splitter, PA polarization analyzer. **b** Experimental results illustrating measured polarization states represented within the Poincaré sphere. **c**–**e** Measured SoP distributions within the Poincaré sphere for samples 6, 11, and 15, respectively. Insets show corresponding scanning electron microscope (SEM) images of each metasurface. **f**–**h** Experimentally measured Stokes parameters for samples 6, 11, and 15, respectively. **i** Calculated Stokes Euclidean Distances (SED) for all fabricated metasurfaces

Experimental validation of the proposed approach is presented in Fig. 4. Figure 4a illustrates the experimental setup, where a polarization scrambler (PS) was employed to generate randomly polarized light, simulating natural light input with a modulation frequency of 2 Hz. The polarization analyzer operated at a sampling rate of 15 Hz, significantly exceeding the Nyquist sampling frequency, ensuring accurate measurement of the output polarization states. Detailed information on the experimental setup is provided in the “Materials and methods” section.

In our experiment, we fabricated and measured 15 samples, each specifically designed to achieve a unique and uniform full-Stokes polarization conversion, encompassing both the SoP and DoP. Among them, 8 samples were designed to demonstrate polarization evolution along the latitude on the Poincaré sphere, with a step size of 45°. Three samples were used to illustrate changes along the longitude, covering linear polarization (LP), elliptical polarization (EP), and right-handed circular polarization (RCP). The remaining 4 samples were dedicated to demonstrating DoP control. All samples were fabricated using a standard CMOS-compatible process, with details provided in the “Materials and methods” section.

Representative experimental results from each of the three groups, specifically samples No. 6, 11, and 15, are presented in Fig. 4. Additional data, arranged sequentially, can be found in Supplementary Information Note VII. Figure 4c–e illustrates the distribution of the experimentally measured polarization states on the Poincaré sphere for three selected samples, accompanied by the corresponding SEM images of the fabricated metasurfaces. The selected samples correspond to polarization states with parameters 2ψ = 225°, 2χ = 90°, and DoP = 0.9, respectively. Figure 4f–h depicts the measured Stokes parameters for all polarization states in each sample. Based on the theoretical analysis, the distribution of partially polarized light on the Poincaré sphere is expected to show a clustering effect around the dominant polarization state as the DoP increases. This trend is confirmed by the experimental results, where the target Stokes parameters are marked with white dashed lines. The measured polarization states exhibit a higher density of points near the dominant polarization state, supporting the theoretical predictions.

The accuracy of the partially polarized light conversion is quantified by the Stokes Euclidean Distance (SED), which is defined as 1−distance, where “distance” represents the Euclidean distance between the measured and target Stokes vectors. A value of SED = 1 indicates perfect alignment (i.e., zero Euclidean distance) between the two Stokes vectors. Figure 4i presents the SED values for all 15 experimental samples, with an average SED of 0.90, compared to the theoretical simulation average of 0.93. The accuracy of experimental results for the three representative groups of samples is measured as 0.942, 0.901, and 0.969, respectively, demonstrating the reliability of our proposed method.

To further demonstrate the feasibility and flexibility of this approach, we showcase the ability to generate different partially polarized light using the same metasurface arrangement. Since the coherent pixels operate in a weak coupling regime, small variations in the gap distance between adjacent meta-atoms have a negligible effect on the modulation behavior. This introduces a tolerance in the gap distance, allowing the same metasurface design to be applied across different modulation scenarios.

**Fig. 5:**
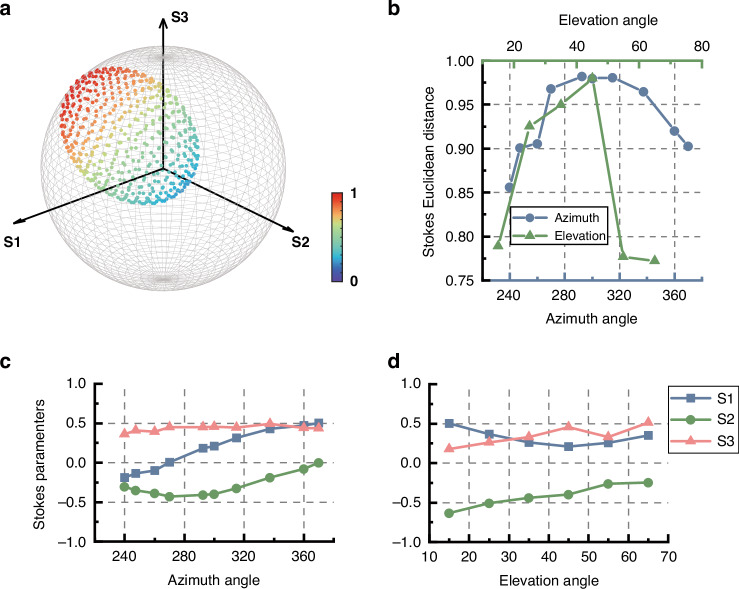
**The robustness of the generalized lattice-based arrangement approach.** **a** Distribution of all polarization states of partially polarized light (2*ψ* = 300°, 2*χ* = 45°, DoP = 0.65) in the time domain on a solid Poincaré sphere. The color represents the light intensity and is normalized. **b** Robustness of metasurface design based on the generalized lattice approach. Taking the position of partially polarized light represented by (**a**) on the Poincaré sphere as the origin, the metasurface is constructed using the same layout along the longitude and latitude directions. The intersection of the two lines is the state represented by (**a**). **c**, **d** Stokes parameters of the selected points in (**b**)

For instance, the layout is designed to achieve a DoP of 0.65, with a quantity ratio of 2.714, as previously illustrated in Fig. 2c. In this layout, the effective sizes of the meta-atoms are (850, 650) nm and (760, 850) nm. The dominant polarization state is initialized at 2*ψ* = 300° and 2*χ* = 45°. The distribution of SoP at this point is shown in Fig. 5a, obtained through numerical simulations. The corresponding SED is calculated to be 0.989, confirming the high accuracy of the designed metasurface in generating the target polarization state. Starting from the initial point, we varied the sizes and rotation angles of the meta-atoms, causing the partially polarized light to move along both the longitude and latitude lines passing through the initial point.

Figure 5b illustrates the operational range of the disordered metasurface, defined as the effective polarization conversion range in terms of latitude and longitude on the Poincaré sphere. Stokes parameters ${S}_{1}-{S}_{3}$ are also given for each point in Fig. 5c, d. In Fig. 5b, the intersection of these two lines represents the initial point. The tolerance in the azimuthal angle (meta-atom rotation) is approximately 60° (with the range of SED exceeding 0.95), while the elevation angle (meta-atom size) tolerance is less than 10°. This discrepancy arises because adjusting the elevation angle requires a complete change in the meta-atom combination, for which the initial effective sizes are not suitable. Although the physical gap between different meta-atom combinations may exhibit varying tolerances, the specific values presented here are not universal. Nonetheless, these results confirm that the metasurface design provides a tolerance margin, allowing the same layout to be used for different meta-atom configurations. This tolerance is crucial for practical applications, as it enables flexible and independent control of both the DoP and SoP.

## Discussion

In the article, we propose a disordered metasurface based on the generalized lattice approach that converts unpolarized light into partially polarized light, achieving independent control over both the SoP and DoP. This approach harnesses far-field interference between meta-atoms and introduces a novel design parameter—the meta-atom quantity ratio—to enable arbitrary and flexible manipulation of polarization states. By generalizing this method, we derive an analytical solution that directly maps the optical parameters of meta-atoms to each element of an arbitrary Jones matrix, as detailed in Supplementary Information Note VIII using a triatomic design. Unlike inverse design methods^32^, our approach offers a more intuitive insight into the underlying physics, establishing a clear and concise relationship between meta-atom properties, the Jones matrix, and full Stokes parameter control. In contrast to traditional placement strategies, such as periodic arrangements that introduce strong higher-order diffraction or interleaved designs^49^ that result in crosstalk, our approach ensures uniformity and coherence by globally arranging meta-atoms based on a generalized lattice.

This work establishes an analytical relationship between nanostructure parameters and polarization and demonstrates the physical realization of partially polarizing natural light through the introduction of the meta-atom quantity ratio. This clear relationship enables designers to directly derive the required nanostructure parameters from target polarization states, reducing reliance on complex numerical optimization methods such as inverse design. The additional degrees of freedom introduced by the generalized lattice design, particularly through the meta-atom quantity ratio, greatly enhance the flexibility of polarization control, surpassing conventional techniques and providing new insights into the physics of non-periodic and asymmetric structures. In addition, the design based on the weak coupling region further improves the robustness of the generalized lattice framework. The working range of the weak coupling region is also validated in Supplementary Information Note IX. This advancement allows for the independent tuning of all Stokes parameters, unlocking opportunities for advanced photonic applications that require precise and independent polarization control, and paving the way for next-generation systems capable of sophisticated multi-dimensional light manipulation.

## Materials and methods

### Fabrication of the disordered metasurface

The metasurface was fabricated on a commercially available 940-nm-thick α-Si layer (PECVD) deposited on a sapphire substrate. The structure was patterned on a ZEP520A resist using an E-beam writer (Raith E-line, 50 kV, 20 nA). After developing the resist, the pattern was transferred onto a 100-nm-thick chromium film. Subsequently, the silicon layer was etched using inductively coupled deep reactive ion etching (DRIE), with the chromium layer serving as a hard mask. The resist was then removed with acetone, followed by rinsing with IPA and DI water. Finally, the remaining chromium mask was removed using a chromium etchant.

### Experimental setup

The experimental setup is designed to precisely control and analyze the polarization state of light interacting with the sample. A polarization scrambler (Luna PSY 201) is used to control the input polarization state, ensuring a well-defined and tunable polarization condition. The optical path begins with Objective 1 (10X, NA = 0.17), which functions as a condenser lens to focus the incident light near the sample. Objective 2 (50X Mitutoyo Plan Apo NIR Infinity Corrected Objective, NA = 0.42) is then used for light collection and microscopic imaging of the sample. A 4f imaging system, composed of a tube lens and a Fourier lens, is implemented to relay the optical field while maintaining spatial coherence. At the conjugate sample plane within this system, an iris diaphragm is placed to block unwanted light from outside the sample area, improving signal quality. An imaging lens is used to project the sample image onto a CCD camera, allowing direct observation and adjustment of the iris diaphragm size. The polarization state of the transmitted light is analyzed using a polarization analyzer (Thorlabs PAX1000IR2).

## Supplementary information

Supplemental Information

## Data Availability

The data that support the findings of this study are available from the corresponding author upon reasonable request.
