# Supplementary material for: Decoupling metasurface parameters for independent Stokes polarization control via generalized lattice
Source: Light Sci Appl. 2026 Jan 4;15:33. doi: 10.1038/s41377-025-02084-6 (PMC12764457; doi:10.1038/s41377-025-02084-6)
Supplement: Supplementary file 1 — Supplemental Information [file 41377_2025_2084_MOESM1_ESM.docx]

Supplemental Information for

**Decoupling Metasurface Parameters for Independent Stokes Polarization Control via Generalized Lattice**

*Zhi Cheng^1^, Zhou Zhou^2^, Zhuo Wang^3^, Yue Wang^1^, Changyuan Yu ^1*^*

*^1^ Department of Electrical and Electronic Engineering,*

*the Hong Kong Polytechnic University, Hong Kong SAR, China*

*^2^ Department of Electrical and Computer Engineering, and NUS Graduate School,*

*National University of Singapore, Singapore 117583, Singapore*

*^3^ Guangdong Provincial Key Laboratory of Nanophotonic Functional Materials and Devices, School of Optoelectronic Science and Engineering,*

*South China Normal University 510006, Guangzhou, China*

** Corresponding Author*

*changyuan.yu@polyu.edu.hk*

Tel: **+**852 2766 6258

Note I: Coupling Between Dominant State of Polarization and Degree of Polarization in diatomic design

Under the weak-coupling regime, the Jones matrix of the diatomic metasurface can be expressed as the sum of each meta-atom’s Jones matrix. This means that arbitrary Jones matrices can be constructed using this approach. The summation process of the Jones matrix elements is illustrated in Fig. S1: the small blue dashed circle represents the unit vector, indicating the complex amplitude of the combined Jones matrix elements. The large blue region, with a modulus of 2, represents the total magnitude of the combined vectors.

The initial combined vector, shown as the green arrow with an amplitude of $A_{1}$, can be decomposed into two unit vectors. When attempting to adjust $A_{1}$ *to* $A_{1}^{'}$ —that is, when trying to change the polarization conversion properties—simply adjusting the surface parameters results in a new combined vector $A_{1}^{'}$ (yellow arrow). However, we observe that the new unit vectors differ substantially from the original ones. This indicates that in the design of diatomic metasurfaces for polarization control, adjustments to polarization conversion properties affect all variables, and modifying just one or a few parameters will not suffice to achieve the desired results.

In other words, practical design requires finding new combinations of meta-atoms and adjusting rotation angles and other parameters to meet the design requirements.


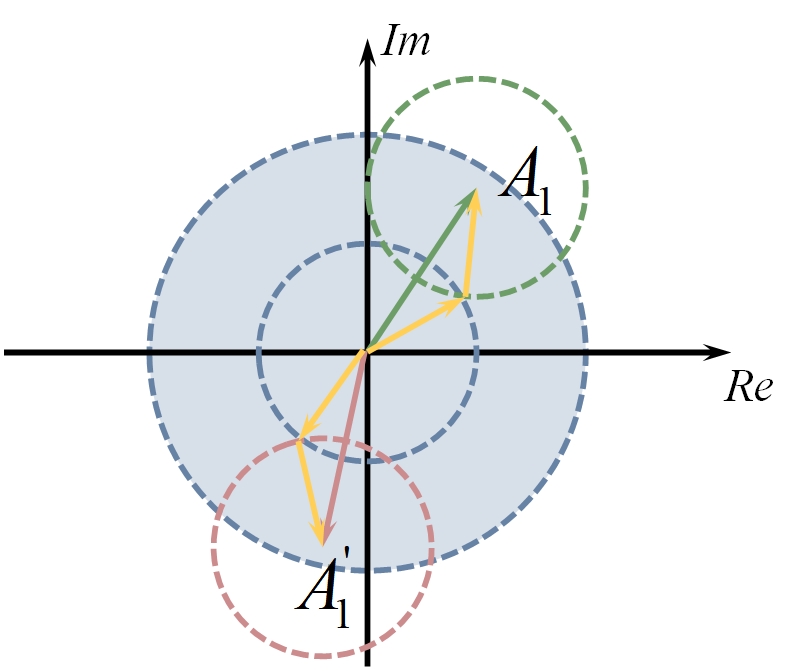


Fig. S1. Phasor diagram of the summation process of Jones matrix elements.

Note II: Derivation of the Jones Matrix for Independent Control of SoP and DoP

Given an arbitrary elliptical polarization state, the states ***α*** and ***β*** are expressed as:

$\alpha=R(\psi-45^{\circ})\left(\begin{matrix} e^{-i\chi} \\ e^{i\chi} \end{matrix}\right),\beta=R(\psi-45^{\circ})\left(\begin{matrix} e^{-i\chi} \\ -e^{i\chi} \end{matrix}\right)$ （S1）

The corresponding conjugate polarization states, ***α**** and ***β****, are represented as ：

$\alpha^{*}=R(\psi-45^{\circ})\left(\begin{matrix} e^{i\chi} \\ e^{-i\chi} \end{matrix}\right),\beta^{*}=R(\psi-45^{\circ})\left(\begin{matrix} e^{i\chi} \\ -e^{-i\chi} \end{matrix}\right)$ (S2)

We aim to find a Jones matrixes J that either convert (***α****, ***β****) into (***α***, ***β***) or (***α***, ***βe^iπ^***). Thus, we seek：

$\begin{matrix} J_{1}(a^{*},\beta^{*})=(\alpha,\beta) \\ J_{2}(a^{*},\beta^{*})=(\alpha,\beta e^{-i\pi}) \end{matrix}$ （S3）

Noting that the Jones matrix for this system, as shown in previous works, must involve a rotation matrix combined with a diagonal matrix, we first rotate the Jones matrix in alignment with the elliptical polarization states, which corresponds to a rotation by *ψ*-45°：

$J=R(\psi-45^{^{\circ}})DR^{-1}(\psi-45^{^{\circ}})$ （S4）

where the matrix D is of the form：

$D=\left(\begin{matrix} d_{11} & 0 \\ 0 & d_{22} \end{matrix}\right)$ (S5)

Substituting Eq. (S4) into the first Eq. of (S3) yields:

$J\alpha^{*}=R(\psi-45^{^{\circ}})DR^{-1}(\psi-45^{^{\circ}})R(\psi-45^{^{\circ}})\left(\begin{matrix} e^{i\chi} \\ e^{-i\chi} \end{matrix}\right)=R(\psi-45^{^{\circ}})D\left(\begin{matrix} e^{i\chi} \\ e^{-i\chi} \end{matrix}\right)=\alpha$ (S6)

It is found that for *J_1_*, the same equation is obtained for both ***α*** and ***β***. Since *R* is an invertible matrix, the above equation can be simplified to:

$D\left(\begin{matrix} e^{i\chi} \\ e^{-i\chi} \end{matrix}\right)=\left(\begin{matrix} e^{-i\chi} \\ e^{i\chi} \end{matrix}\right)$ (S7)

Solving the equations, we get：

$\begin{matrix} d_{11}e^{i\chi}=e^{-i\chi} \Rightarrow d_{11}=e^{-2i\chi} \\ d_{22}e^{-i\chi}=e^{i\chi} \Rightarrow d_{22}=e^{2i\chi} \end{matrix}$ (S8)

Thus, the required Jones matrix is:

$J_{1}=R(\psi-45^{^{\circ}})\left(\begin{matrix} e^{-2i\chi} & 0 \\ 0 & e^{2i\chi} \end{matrix}\right)R^{-1}(\psi-45^{^{\circ}})$ (S9)

It is easy to see that in order to find a suitable *J_2_*, if the Jones matrix continues to rotate by *ψ*-45°, substituting Eq. (S4) into the second term of Eq. (S3) yields no solution. Therefore, different rotation angles need to be considered by solving the following system of equations:

$\begin{matrix} DR(\theta)\left(\begin{matrix} e^{i\chi} \\ e^{-i\chi} \end{matrix}\right)=R(\theta)\left(\begin{matrix} e^{-i\chi} \\ e^{i\chi} \end{matrix}\right) \\ DR(\theta)\left(\begin{matrix} e^{i\chi} \\ -e^{-i\chi} \end{matrix}\right)=R(\theta)\left(\begin{matrix} -e^{-i\chi} \\ e^{i\chi} \end{matrix}\right) \end{matrix}$ (S10)

Where *θ* = *ψ* - 45° - *ψ*₂, with *ψ*₂ being the rotation angle of *J_2_*. Expanding *R(θ)* gives the following two sets of equations:

$\begin{matrix} \left\{\begin{matrix} d_{11}\cos\theta\cdot e^{i\chi}-d_{11}\sin\theta\cdot e^{-i\chi}=e^{-i\chi}\cos\theta-e^{i\chi}\sin\theta\\ d_{22}\sin\theta\cdot e^{i\chi}+d_{22}\cos\theta\cdot e^{-i\chi}=e^{-i\chi}\sin\theta+e^{i\chi}\cos\theta\end{matrix}\right. \\ \left\{\begin{matrix} d_{11}\cos\theta\cdot e^{i\chi}+d_{11}\sin\theta\cdot e^{-i\chi}=-e^{-i\chi}\cos\theta-e^{i\chi}\sin\theta\\ d_{22}\sin\theta\cdot e^{i\chi}-d_{22}\cos\theta\cdot e^{-i\chi}=e^{-i\chi}\sin\theta+e^{i\chi}\cos\theta\end{matrix}\right. \end{matrix}$ (S11)

By comparing the exponents of the exponential terms on both sides of the equation, the following relationships can be derived:

$\begin{matrix} \left\{\begin{matrix} d_{11}\cos\theta=-\sin\theta\\ d_{11}\sin\theta=-\cos\theta\end{matrix}\right. \\ \left\{\begin{matrix} d_{22}\sin\theta=\cos\theta\\ -d_{22}\cos\theta=\sin\theta\end{matrix}\right. \end{matrix}$ (S12)

In order for the above equations to hold, *d_i_* (*i* = 1, 2) must satisfy the following conditions:

$\left\{\begin{matrix} d_{1}=-\tan\theta=-\cot\theta\\ d_{2}=\tan\theta=\cot\theta\end{matrix}\right.$ (S13)

Then we get:

$\theta={45}^{\circ}+n\cdot{90}^{\circ}, n\in N$ (S14)

Let $\theta$ = -45°，we get *ψ*_2_ = *ψ*. Thus, we get *d*_1_ = 1 and *d_2_* = -1, with the Jones matrix in the following form:

$J_{2}=R(\psi)\left(\begin{matrix} 1 & 0 \\ 0 & -1 \end{matrix}\right)R^{-1}(\psi)$ (S15)

Thus, the Jones matrix that accounts for the difference conversion between an orthogonal polarization pair can be written as:

$J=J_{1}+J_{2}= \frac{n_{1}}{N}R(\psi-45^{\circ})\left(\begin{matrix} e^{-2i\chi} & 0 \\ 0 & e^{2i\chi} \end{matrix}\right)R(45^{\circ}-\psi)+\frac{n_{2}}{N}R(\psi)\left(\begin{matrix} 1 & 0 \\ 0 & -1 \end{matrix}\right)R(-\psi)$ (S16)

Note III: The Mueller matrix of the proposed disordered metasurface

The following derivation explains the transformation from the Jones matrix ***J*** to the Mueller matrix ***M***, which is essential for describing the polarization conversion process of partially polarizing unpolarized light. The general form of the Mueller matrix ***M*** is expressed as:

$M_{0}=\left[ \begin{matrix} m_{00} & m_{01} & \cdots& m_{04} \\ \vdots& \vdots& \ddots& \vdots\\ m_{30} & \cdots& \cdots& m_{33} \end{matrix} \right]$ (S17)

Since the Stokes parameters of unpolarized light are $S_{UP}=\left[ 1,0,0,0 \right]^{T}$, only the first column of $M_{\boldsymbol{0}}$needs to be calculated.

The relationship between the Jones matrix $J$ and the Mueller matrix $M$ is defined as:

$M=U\cdot\left( J\otimes J^{*} \right)\cdot U^{-1}$ (S18)

Where $\otimes$ denotes the Kronecker product, and the matrix ***U*** is defined as:

$U=\frac{1}{2}\left[ \begin{matrix} 1 & 0 & 0 & 1 \\ 1 & 0 & 0 & -1 \\ 0 & 1 & 1 & 0 \\ 0 & i & -i & 0 \end{matrix} \right]$ (S19)

The Jones matrix in Eq.(S16) can be written as:

$J\frac{n_{1}}{N}R\left( \psi-{45}^{\circ} \right)\left[ \begin{matrix} e^{-2i\chi} & \frac{n_{2}}{n_{1}} \\ \frac{n_{2}}{n_{1}} & e^{2i\chi} \end{matrix} \right]R=\left( {45}^{\circ}-\psi\right)=R\left( \theta\right)\left[ \begin{matrix} A & B \\ C & D \end{matrix} \right]R\left( -\theta\right)$ (S20)

To simplify, we first calculate $M_{1}=U\left( J_{1}^{*}\otimes J_{1} \right)U^{-1}$*, where* $J_{1}=\left[ A,B;C,D \right].$ Using the definition of the Kronecker product, $J \otimes J^{*}$can be written as:

$J\otimes J^{*}=\left[ \begin{matrix} B^{2}+\frac{1}{2}\left( DD^{*}+AA^{*} \right) & \cdots& \cdots& \cdots\\ \frac{1}{2}\left( AA^{*}-DD^{*} \right) & \cdots& \cdots& \cdots\\ \frac{1}{2}\left( BA^{*}+BD^{*}+BA+BD \right) & \cdots& \cdots& \cdots\\ \frac{i}{2}\left( BA^{*}-BD^{*}-BA+BD \right) & \cdots& \cdots& \cdots\end{matrix} \right]$ (S21)

*With* $A=D^{*}$*,* the matrix $M_{1}$ simplifies to:

$M_{1}=\left[ \begin{matrix} B^{2}+1 & \cdots& \cdots& \cdots\\ D & \cdots& \cdots& \cdots\\ B\left( A+D \right) & \cdots& \cdots& \cdots\\ Bi\left( -A+D \right) & \cdots& \cdots& \cdots\end{matrix} \right]\underset{\to}{Normlization}\left[ \begin{matrix} 1 & \cdots& \cdots& \cdots\\ 0 & \cdots& \cdots& \cdots\\ \frac{B}{1+B^{2}}\left( A+D \right) & \cdots& \cdots& \cdots\\ \frac{Bi}{1+B^{2}}\left( D-A \right) & \cdots& \cdots& \cdots\end{matrix} \right]$ (S22)

Next, we consider the general form of the Mueller matrix $M_{0}$ under rotation, defined as:

$M\left( \theta\right)M_{0}M\left( -\theta\right)=\left[ \begin{matrix} m_{00} & \cdots& \cdots& \cdots\\ m_{10}\cos2\theta+m_{20}\sin2\theta& \cdots& \cdots& \cdots\\ m_{20}\cos2\theta-m_{10}\sin2\theta& \cdots& \cdots& \cdots\\ m_{30} & \cdots& \cdots& \cdots\end{matrix} \right]$ (S23)

By substituting $\theta=\psi-{45}^{\circ}$ into the above equation and using Eq. (S22), the final Mueller matrix is expressed as:

$M=M\left( \theta\right)M_{1}M\left( -\theta\right)=\left[ \begin{matrix} 1 & \cdots& \cdots& \cdots\\ p\cos2\psi\cos2\chi& \cdots& \cdots& \cdots\\ p\sin2\psi\cos2\chi& \cdots& \cdots& \cdots\\ p\sin2\chi& \cdots& \cdots& \cdots\end{matrix} \right]$ (S24)

Where $p=\frac{2B}{1-B^{2}}$ denotes to the DoP.

Note IV: The necessity of introducing Disorder compared to Periodic construction

Although it may seem intuitive to use a periodic supercell construction to achieve the Jones matrix presented in Eq. 9 of the main text, several potential issues arise when applying periodic designs to this task. Consider the target polarization conversion parameters stated in the main text, with 2$\psi$ = ${300}^{\circ}$, 2$\chi$ = ${45}^{\circ}$, and DoP = 0.65. The dimensions of the two meta-atoms involved are 320 nm by 650 nm and 600 nm by 250 nm, respectively. One might assume that arranging these meta-atoms uniformly in a supercell, as illustrated in the inset of the Fig. S2a, would yield the desired result. An 8:3 ratio of meta-atoms is used to approximate the target ratio of 2.743, which is required for the desired DoP control. However, due to the periodic boundary conditions, when the supercell is translated, the meta-atom at the bottom-left corner (meta-atom B) aligns with the meta-atom at the top-right corner of the adjacent supercell. This leads to an excess of meta-atoms B in certain local regions, potentially diminishing the polarization conversion efficiency. This perturbation effect becomes more pronounced when the number of meta-atoms within the supercell is relatively small. In contrast, if the entire disordered metasurface itself were translationally symmetric, the impact of this perturbation would be negligible. Our simulations for this pattern yield a DoP of 0.714, with the azimuthal and elevation angles of the dominant polarization state at -66.31° and 26.56°, respectively. When evaluated using the Euclidean distance between Stokes parameters, the similarity to the target result is 0.76, which falls significantly short of the performance achieved using the disordered metasurface design described in the main text.

An alternative approach might be to arrange the meta-atoms within a two-dimensional crystal lattice, such as a rectangular or hexagonal lattice. This can be done by treating a small group of meta-atoms as a unit cell and placing multiple unit cells at lattice vertices. We provide two such examples in Fig. S2b and c. The design in Fig. S2b encounters the same issues as in Fig. S2a, while the design in Fig. S2c generates a quasi-gradient phase along the indicated direction. The white dashed rectangular line represents the larger lattice, while the circular one corresponds to the smaller meta-atom cluster in the Fig. S2b. The stokes similarity of this design is 0.82. Although the periodic arrangement in the inset of Fig. S2c more closely resembles a typical lattice distribution, the strong internal order within the supercell is further enhanced by the periodicity. The arrow in the inset indicates a quasi-phase gradient direction, leading to beam deflection, as confirmed by the far-field intensity shown in Fig. S2c. The computed similarity of the Stokes parameters for this design is only 0.8. Additionally, the periodic structure leads to pronounced higher-order diffraction effects, as the diffraction fields in the Fig. S2 shown.


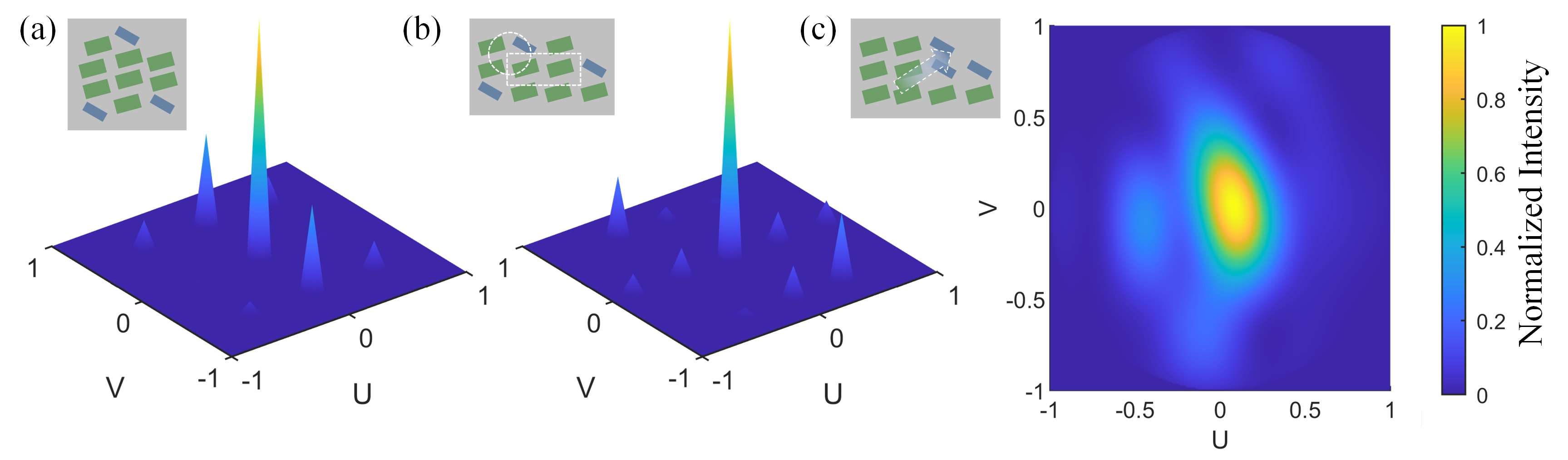


Figure S2. a-b Diffraction field of periodic design. c Intensity distribution of the far-field. Inset: metasurface arrangements for corresponding design. *U* and *V* are the direction cosines.

It is important to note that this discussion does not prove that a disordered metasurface is the only viable solution for achieving this target. Advanced algorithms for periodic design could be developed to address these issues and meet the desired goals. However, directly adopting a disordered design appears to be a more practical approach. Moreover, an appropriate arrangement algorithm (discussed in Note V) is crucial, as it is mathematically impossible to achieve uniform tiling of rectangles with arbitrary proportions and sizes in two dimensions using only simple translations and rotations.

Note V: Flow chart of the algorithm

As stated in the main text, each meta-atom is enclosed by a bounding cell which ensure them working at the weak coupling region. In the home-made algorithm based on 2D bin-packing and greedy heuristic, as illustrated in the Fig. S3, parameters including the quantity ratio, sizes of meta-atoms, and the sizes of the bounding cells are initialized. Candidate placing positions are determined along the arc centered at the origin, which totally forms an outline. The placing position is selected among the candidate positions which is the one located nearest to the origin.

Fitness is defined as a measure of how closely the area ratios of different rectangle types within local regions of the plane match their ideal proportions, which are based on the given quantities of each rectangle type. The fitness function can be quantized by the following formula:

$$\text{Fitness}=\frac{1}{N\times M}\sum_{\text{grid}} \left( \frac{1}{n_{\text{atoms}}}\sum_{\text{atoms}} \frac{\left| \eta_{\text{measure,new}}-\eta_{\text{target}} \right|}{\left| \eta_{\text{measure,old}}-\eta_{\text{target}} \right|} \right)$$

where$\eta_{\text{measure,old}}$ and $\eta_{\text{measure, new}}$ are the measured proportions of meta-atoms within the local evaluation region before and after placement, respectively. $\eta_{\text{target}}$ is the target proportion of meta-atoms. $n_{\text{atoms}}$ is the number of meta-atoms in the local evaluation region. Over the whole metasurface, a grid with $N \times M$ nodes is applied to calculate the average fitness value. At each step, only the placement that provides the maximum local improvement in fitness is chosen, without considering the global impact of this decision. The algorithm focuses on immediate, local uniformity while ignoring potential long-term effects on the overall layout. This ensure the effecitive of this home-made algorithm. Additionally, new rectangles are placed along the current outline to prioritize dense packing, further emphasizing the greedy, short-term optimization approach.


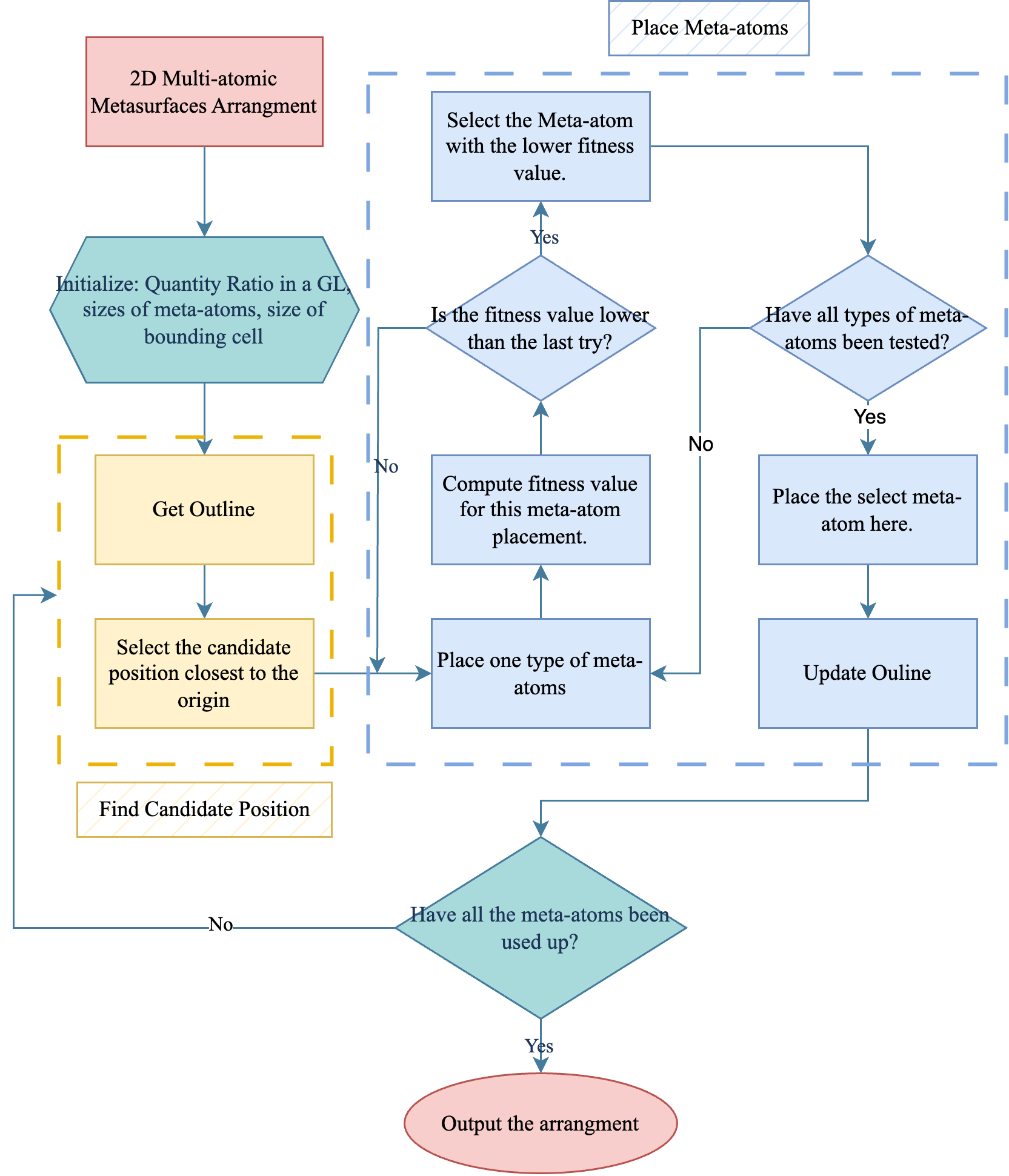


Figure S3. The flow chart of the arrangement algorithm. GL: generalized lattice.

Note VI: Original structural and arrangement parameters for the main text results

We constructed a nanostructure database consisting of rectangular silicon (*RI* = 3.45 @ 1550 nm) nanopillars on a silica substrate (*RI* = 1.45 @ 1550 nm). The thickness of the nanopillars is 940 nm, and the period during parameter sweeping is set to 960 nm. The periodic boundary condition is applied in the simulation. The length and width are varied between 200 and 800 nm, as illustrated in Fig. S4. The meta-atoms used to demonstrate the evolution along the latitude on the Poincaré sphere have dimensions of 320 nm by 650 nm and 600 nm by 250 nm. The arrangement data for 2*ψ*=300∘ is provided in the table S1 and S2. The effective sizes of these meta-atoms are 850 nm by 650 nm and 760 nm by 850 nm, respectively. Table TS1 lists the position coordinates of meta-atoms A, while Table TS2 provides the coordinates for meta-atoms B. For the evolution along the longitude on the Poincaré sphere, the data includes the size parameters for each set of meta-atom combinations at each longitude, which are listed in Table S3. For DoP modulation, the meta-atoms have dimensions of 220 nm by 590 nm and 690 nm by 250 nm, respectively.

The other raw data that support the findings of this study are available from the corresponding author upon reasonable request.

Table S1. Position coordinates of meta-atom with dimensions (320, 650) nm

| *X* | *Y* | *X* | *Y* | *X* | *Y* | *X* | *Y* | *X* | *Y* |
| --- | --- | --- | --- | --- | --- | --- | --- | --- | --- |
| 425 | 325 | 1275 | 325 | 1185 | 975 | 425 | 1825 | 1275 | 1625 |
| 2125 | 1175 | 425 | 2475 | 2275 | 1825 | 3125 | 325 | 575 | 3125 |
| 2065 | 2475 | 3125 | 975 | 1425 | 3125 | 2915 | 2475 | 2275 | 3125 |
| 1195 | 3775 | 3975 | 1175 | 3885 | 1825 | 3775 | 2475 | 1195 | 4425 |
| 4825 | 325 | 2805 | 3975 | 4825 | 975 | 2045 | 4625 | 4735 | 1825 |
| 4005 | 3125 | 425 | 5075 | 3655 | 3975 | 1275 | 5275 | 2895 | 4625 |
| 5675 | 325 | 4505 | 3775 | 5615 | 1825 | 2885 | 5275 | 1185 | 5925 |
| 5615 | 2475 | 5355 | 3325 | 4505 | 4425 | 2035 | 6125 | 6525 | 325 |
| 425 | 6575 | 2885 | 5925 | 6525 | 975 | 3735 | 5475 | 6465 | 1625 |
| 4585 | 5075 | 6465 | 2275 | 1275 | 6775 | 6205 | 3125 | 2885 | 6575 |
| 425 | 7225 | 5445 | 4825 | 4585 | 5725 | 6295 | 3775 | 7315 | 1625 |
| 6315 | 4425 | 1335 | 7625 | 2885 | 7225 | 4495 | 6375 | 7165 | 3125 |
| 3735 | 7025 | 6295 | 5075 | 8165 | 325 | 2185 | 7875 | 8165 | 975 |
| 5345 | 6375 | 8165 | 1625 | 1205 | 8275 | 4585 | 7025 | 8095 | 2275 |
| 3035 | 7875 | 6215 | 5725 | 7195 | 4625 | 8095 | 2925 | 2055 | 8525 |
| 8045 | 3575 | 6215 | 6375 | 425 | 8925 | 9035 | 1175 | 8085 | 4225 |
| 4655 | 7875 | 3415 | 8525 | 8945 | 2675 | 7065 | 6125 | 6195 | 7025 |
| 8045 | 4875 | 2115 | 9175 | 4265 | 8525 | 425 | 9575 | 5505 | 7875 |
| 2965 | 9175 | 7915 | 5525 | 8935 | 4175 | 9885 | 325 | 1275 | 9825 |

Table S2. Position coordinates of meta-atom with dimensions (600, 250) nm.

| *X* | *Y* | *X* | *Y* | *X* | *Y* | *X* | *Y* | *X* | *Y* |
| --- | --- | --- | --- | --- | --- | --- | --- | --- | --- |
| 380 | 1075 | 2080 | 425 | 1230 | 2375 | 3080 | 1725 | 390 | 3875 |
| 3930 | 425 | 2000 | 3875 | 3200 | 3225 | 4810 | 2575 | 5630 | 1075 |
| 2080 | 5375 | 380 | 5825 | 3700 | 4725 | 5310 | 4075 | 2080 | 6875 |
| 3690 | 6225 | 7330 | 425 | 7290 | 2375 | 5410 | 5575 | 400 | 7975 |
| 7120 | 3875 | 3850 | 7775 | 7100 | 5375 | 5390 | 7125 | 9030 | 425 |
| 1250 | 9025 | 8970 | 1925 | 8900 | 3425 | 7020 | 6875 | 5100 | 8625 |
| 8890 | 4925 | 2380 | 9925 | 380 | 10325 | 10400 | 1075 | 8470 | 6275 |

Table S3. Structure parameters for demonstrating polarization evolution along a longitude on the Poincaré sphere.

| 2*χ* | -90 | -67.5 | -45 | -22.5 | 0 | 22.5 | 45 | 67.5 | 90 |
| --- | --- | --- | --- | --- | --- | --- | --- | --- | --- |
| Meta-atom A (L,W) nm | 470,250 | 240,510 | 340,750 | 560,370 | 550,550 | 360,520 | 320,650 | 240,500 | 250,470 |
| Meta-atom B (L,W) nm | 250,680 | 680,250 | 250,760 | 610,250 | 690,250 | 580,250 | 600,250 | 250,690 | 250,680 |


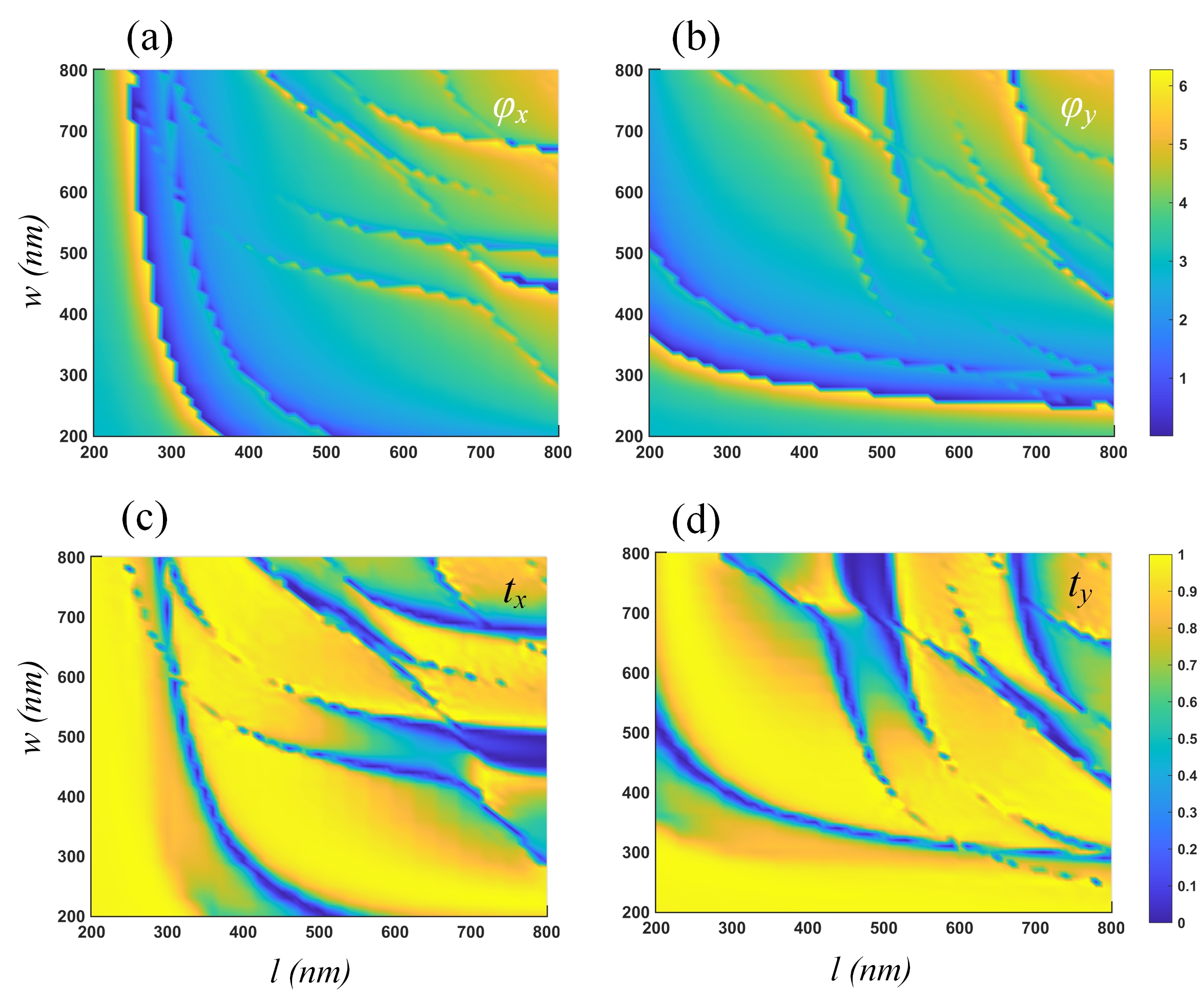


Figure S4 a-b. Phase delay and c-d transmission coefficients with respect to the meta-atoms’ length (l) and width (w) at a wavelength of 1550 nm for x-polarized and y-polarized incident light.

Note VII: Experimental results

The measurement results for all fabricated samples are presented in the Fig. S5. The first and second rows illustrate the evolution of the polarization state along the latitude lines of the Poincaré sphere, corresponding to variations in 2*ψ* from 0° to 315°. The third row represents the evolution along the longitude lines, covering different polarization states, including linear polarization (LP), elliptical polarization (EP), and right circular polarization (RCP). The fourth row demonstrates the variation in the degree of polarization (DoP), with values of 0.15, 0.4, 0.65, and 0.9.

For each measurement, the inset in the lower right corner of each figure displays the scanning electron microscope (SEM) image of the corresponding fabricated sample, providing a structural reference for the observed polarization characteristics.





Figure S5. Experimental results of all fabricated samples.

Note VIII: Forward Method for Implementing Arbitrary Jones Matrices

In addition to the modulation described in the main text, the proposed homogeneous disordered metasurface can be applied to realize arbitrary Jones matrices when *M* > 2. While many previous works ^1-6^ have demonstrated that a diatomic design is sufficient to construct arbitrary Jones matrices for single-layer surfaces, the approach of providing more degrees of freedom (DoFs) offers greater flexibility in the selection of meta-atoms, as well as in other design aspects. For an arbitrary Jones matrix, it can be expressed in the following form:

(S25)

The off-diagonal elements of the matrix are symmetric due to mirror symmetry, which is characteristic of single-layer structures ^2^. By applying a 45-degree rotation, the off-diagonal terms can be rotated into the following configuration:

(S26)

The second term can be interpreted as the Jones matrix of a birefringent meta-atom, with fast and slow axes having phase delays of *ϕ*_12_ and *ϕ*_12_+*π*, respectively. The amplitude of *J*_12_ can be retrieved after determining the quantity ratio from the first term. Since the second term has the same form as the Jones matrix of a meta-atom, we only need to decompose the first term. To solve for this decomposition, we can construct the following equation:

(S27)

And we can obtain the following system of equations:

(S28)

We can observe that there are four equations with six variables, indicating that the system has an infinite number of solutions. In this case, it is possible to impose initial conditions on the system to obtain a desired solution. For example, by setting *ϕ*_11_=*ϕ_x_*_1_=*ϕ_y_*_1_, we get *J*_11_ = *x* + *y*. The second equation can then be solved analytically, which can be easily visualized using an Argand diagram shown in Fig. S6a.

As an example, consider constructing a Jones matrix that converts 45° linearly polarized (LP) light to right circularly polarized (RCP) light. The required Jones matrix for this conversion is:

$J=\left(\begin{matrix} 1.2645-0.1285i & -0.2645+0.1285i \\ -0.2645-0.1285i & 0.2645+0.8715i \end{matrix}\right)$ (S29)

Let *x*=*y* and *ϕ* _11_= *ϕ _x_*_1_= *ϕ _y_*_1_. We can then solve for the solution as follows:

(S30)

The corresponding meta-atoms have been selected with dimensions of (730, 240), (220, 290), and (250, 650) nm, respectively, with a quantity ratio of 13:13:6. A portion of the arrangement results is shown in Fig. S6b. To evaluate the accuracy of the polarization conversion, we employ the fidelity, defined as the inner product between the simulated Jones vector and the target Jones vector. The fidelity achieved in this case exceeds 98%.

This example demonstrates that the proposed metasurface and its corresponding construction algorithm can be extended to various configurations of meta-atom combinations. The flexibility in design allows for greater freedom in selecting meta-atoms, and initial conditions can be adjusted according to specific requirements. This design method, with its increased degrees of freedom, allows for a direct mapping between the metasurface's physical parameters and the desired control targets. As a result, the metasurface can be flexibly designed to achieve precise light field manipulation tailored to specific application requirements.


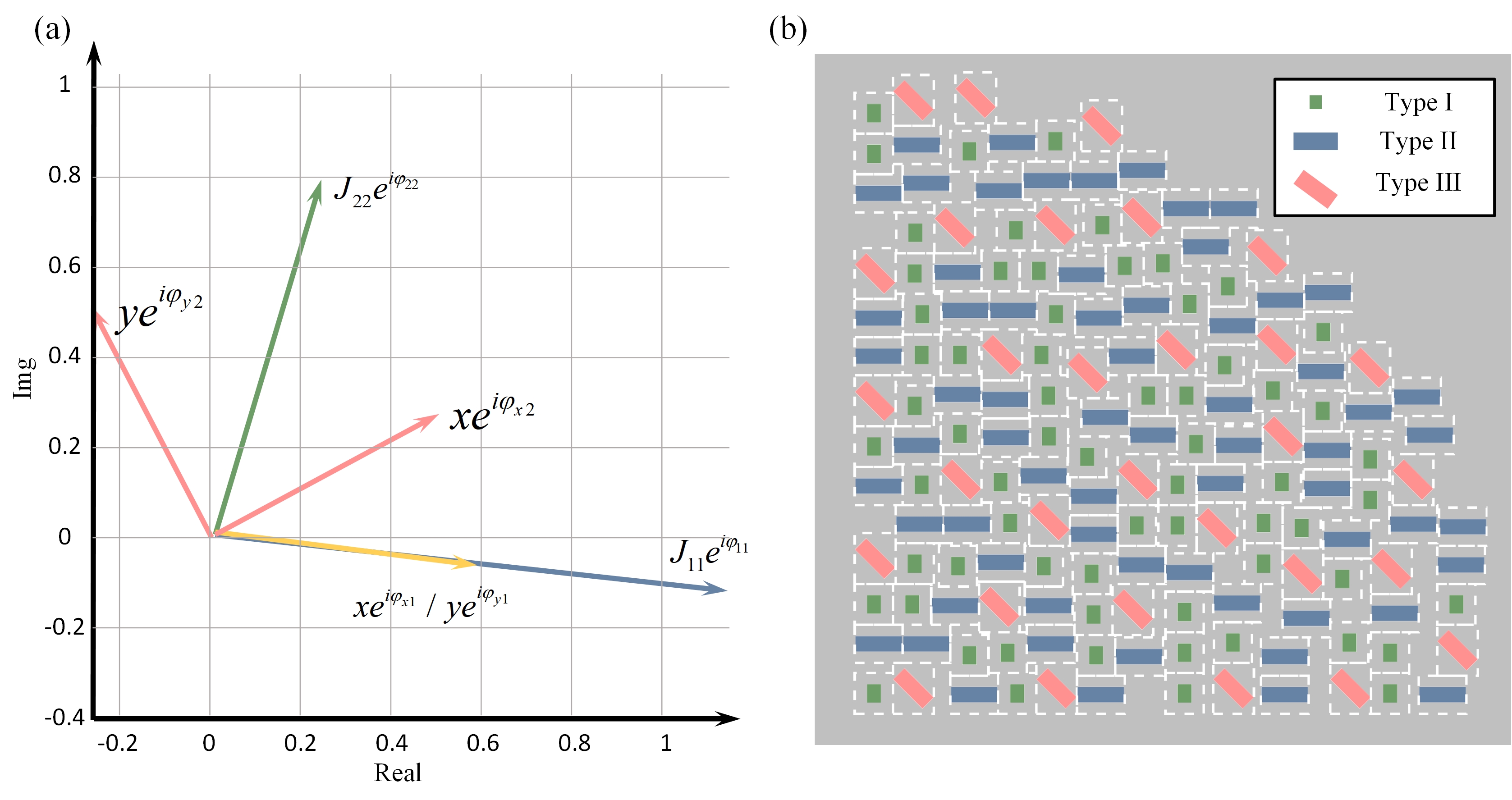


Figure S6. a. Argand diagram to solve the complex equation (Eq. S28). b. Metasurface arrangement of the demonstration for the construction of an arbitrary Jones matrix.

Note IX: Weak coupling region among meta-atoms

Advanced polarization manipulation through far-field interference among multiple meta-atoms requires the spacing between adjacent meta-atoms to lie within the weak coupling region ^5,7^. In this region, the overall Jones matrix can be approximated as the sum of the individual Jones matrices of each meta-atom. If the meta-atoms are placed closer together, stronger coupling occurs, leading to mode coupling or resonance effects. Conversely, if the meta-atoms are positioned farther apart, the phase difference between independently modulated sub-waves cannot be ignored, which also influences the calculation of the Jones matrix.

On the other hand, the weak coupling region typically spans a range, providing greater robustness to the generalized lattice approach we propose. Here, we demonstrate the existence of the weak coupling region and provide reference values for its range under the structural parameters of the meta-atoms discussed in Note VI.

In general, we calculate the propagation phase of a meta-atom by applying periodic boundary conditions, as shown in Figure S4. For the two meta-atoms discussed in Note VI, we varied the periodic boundary size while keeping the atom size constant. As shown in Figures S7(a) and (b), within the shaded gray region, the modulation phase remains nearly unchanged. This demonstrates the existence of the weak coupling region and indicates a certain tolerance when selecting the effective size.


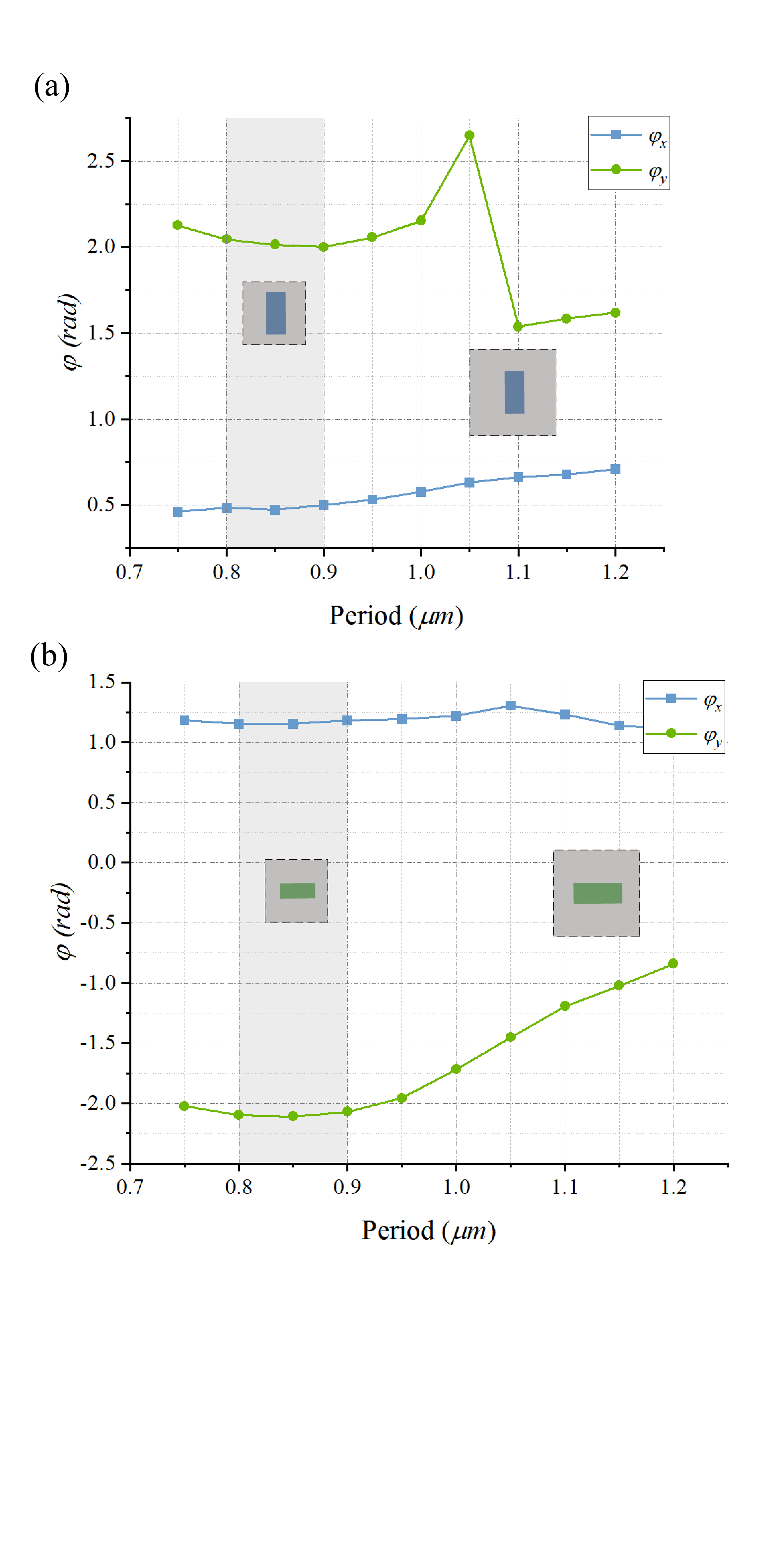


Figure S7. Weak coupling region at the periodic boundary of a single meta-atom. a. meta-atom size: (310, 650) nm. b. meta-atom size: (600, 250) nm.

Additionally, as shown in Figure S8, we considered a diatomic structure. Under x-polarized incident light, we varied the distance between the two meta-atoms. We observed that the Stokes parameters remain nearly constant over a range of approximately 100 nm, further confirming the presence of the weak coupling region.


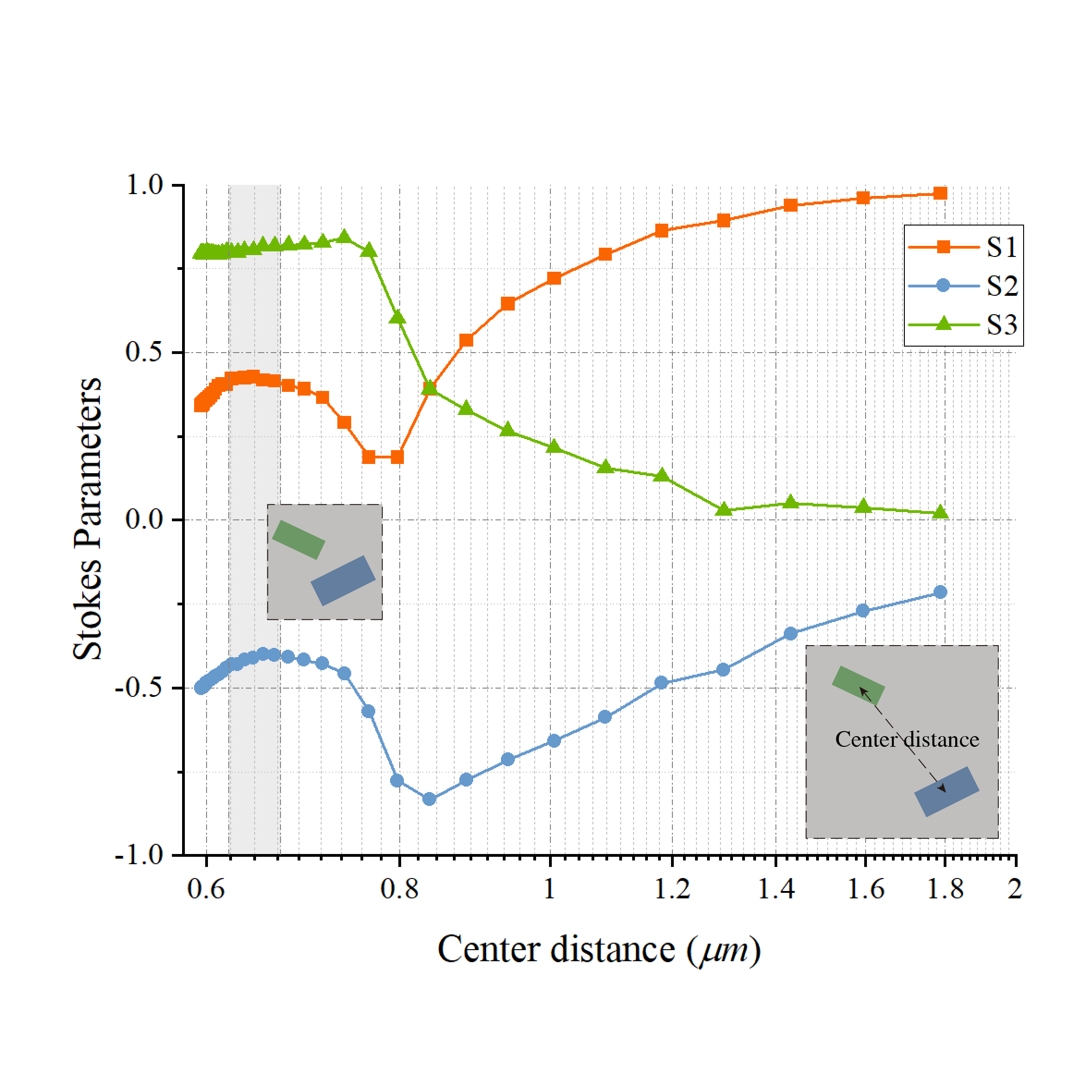


Figure S8. Weak coupling region at the distance between two meta-atoms.

1 Overvig, A. C. *et al.* Dielectric metasurfaces for complete and independent control of the optical amplitude and phase. *Light Sci. Appl.* **8** (2019). https://doi.org/10.1038/s41377-019-0201-7

2 Bao, Y., Wen, L., Chen, Q., Qiu, C.-W. & Li, B. Toward the capacity limit of 2D planar Jones matrix with a single-layer metasurface. *Sci. Adv.* **7**, eabh0365 (2021).

3 Bao, Y., Weng, Q. & Li, B. Conversion between Arbitrary Amplitude, Phase, and Polarization with Minimal Degrees of Freedom of Metasurface. *Laser Photonics Rev.* **16**, 2100280 (2022). https://doi.org/10.1002/lpor.202100280

4 Wu, T. *et al.* Dielectric Metasurfaces for Complete Control of Phase, Amplitude, and Polarization. *Adv. Opt. Mater.* **10**, 2101223 (2022). https://doi.org/10.1002/adom.202101223

5 Feng, C. *et al.* Diatomic Metasurface for Efficient Six‐Channel Modulation of Jones Matrix. *Laser Photonics Rev.* **17**, 2200955 (2023). https://doi.org/10.1002/lpor.202200955

6 Fan, Q. *et al.* Independent Amplitude Control of Arbitrary Orthogonal States of Polarization via Dielectric Metasurfaces. *Phys Rev Lett* **125**, 267402 (2020). <https://doi.org/10.1103/PhysRevLett.125.267402>

7 Bao, Y. et al. Coherent Pixel Design of Metasurfaces for Multidimensional Optical Control of Multiple Printing‐Image Switching and Encoding. Adv. Funct. Mater. 28, 1805306 (2018). https://doi.org/10.1002/adfm.201805306
